# Attachment, Mate-Retention Strategies, and Intimate Partner Violence Victimisation: A Two-Wave Study in Brazil and the United Kingdom

**DOI:** 10.3390/bs16081388

**Published:** 2026-08-12

**Authors:** Bruna Nascimento, Kwanele Shishane, Lora Adair, Nelli Ferenczi, Xiancai Cao, Maame Ama Akwama Ackah, Matthew Callum May

**Affiliations:** 1 School of Psychology & Neuroscience, University of Bristol, Bristol BS8 1TD, UK; xiancai.cao@bristol.ac.uk (X.C.); matt.may2@icloud.com (M.C.M.); 2 School of Society, Community, and Health, University of Bedfordshire, Luton LU1 3JU, UK; kwanele.shishane@beds.ac.uk; 3 Centre for Culture and Evolution, Department of Psychology, Brunel University of London, London UB8 3PH, UK; lora.adair@brunel.ac.uk (L.A.); nelli.ferenczi@brunel.ac.uk (N.F.); 4 Counselling Services, Ashesi University, Accra PMB CT3, Ghana; m.akwamaackah@yahoo.com

**Keywords:** intimate partner violence, attachment, mate-retention, longitudinal, cross-cultural research

## Abstract

The current study examined whether individual differences in attachment orientation, mate-retention tactics (benefit-provisioning and cost-inflicting strategies), and COVID-19-related stressors (impact, uncertainty, and perceived risk) were associated with IPV victimisation across two time points, one month apart, in Brazil and the UK (*N* = 1416 at Wave 1; *N* = 353 at Wave 2). Linear mixed-effects models controlled for age, sex, ethnicity, and relationship length. IPV victimisation did not significantly change over time, and COVID-19-related variables were not associated with any form of IPV. Brazilian participants reported higher psychological IPV than UK participants, although no country differences emerged for physical or sexual IPV. Cost-inflicting mate-retention strategies were positively associated with all forms of IPV in both countries, with consistently stronger associations in the UK. Attachment avoidance was positively associated with all forms of IPV in Brazil but was negatively associated with psychological and sexual IPV in the UK. Attachment anxiety and benefit-provisioning strategies were associated with psychological IPV in Brazil only. These findings suggest that associations between attachment, mate-retention strategies, and IPV victimisation may vary across cultural contexts, underscoring the value of culturally sensitive approaches to IPV research and intervention.

## 1. Introduction

Intimate partner violence (IPV), defined as physical, psychological, or sexual harm perpetrated by a current or former intimate partner (World Health Organization, 2023), remains a pervasive public health problem worldwide. IPV is associated with short- and long-term consequences for physical health and psychological well-being. It has socio-economic consequences for families and negative implications for the development of countries more broadly (Walby & Towers, 2018/2018). IPV disproportionately affects women both in low- and middle-income countries, such as Brazil, where the lifetime prevalence of IPV against women is 18% (Brazil, 2016). In high-income countries, such as the UK, 9% of women and girls, in England and Wales, aged 16 and over, reported having suffered IPV in the year ending March 2022 (Office for National Statistics, 2022). Indeed, the UK government has labelled violence against women and girls as a national threat (Tudor, 2023). Despite the gender disparity seen in IPV, this issue is not limited to women and girls. According to the Centers for Disease Control and Prevention (2024), IPV affects at least 10% of men in their lifetime. Additionally, a meta-analysis of 52 studies (*N* = 32,048) found that the pooled prevalence of IPV amongst gay men is 33% (Liu et al., 2021). IPV victimisation is a problem that affects all genders (Scott-Storey et al., 2022).

Importantly, under circumstances of heightened adversity and social stress, IPV becomes more prevalent. For example, cross-cultural evidence suggests that gender-based violence (GBV) increases during extreme weather and climate events (van Daalen et al., 2022), periods of public health crises (Peitzmeier et al., 2022), and is higher in conflict zones (Kelly et al., 2018). Such events are often associated with economic instability, food scarcity, disrupted infrastructures, and widespread uncertainty, which are established risk factors for IPV (Idriss-Wheeler et al., 2024). As such, COVID-19 may serve as a pertinent context for exploring how widespread stress contributes to IPV risk. Although a substantial body of research has examined IPV during the COVID-19 pandemic, most studies have been cross-sectional and focused primarily on demographic correlates. As a result, important psychological and relational factors have often been overlooked. To address these limitations, the present study draws on the Vulnerability–Stress–Adaptation (VSA) model to investigate how COVID-19-related stressors (e.g., uncertainty, perceived risk), individual differences (e.g., attachment orientation), and relationship-related behaviours predict IPV, with each construct selected to correspond to a distinct component of the model (environmental stress, personal vulnerability, and adaptive relational processes, respectively). It further examines whether these associations differ between Brazil and the United Kingdom, providing a novel test of whether VSA-informed processes generalise across two distinct national contexts or show context-specific patterns that require further investigation.

### Attachment, Mate-Retention, and Pandemic Stress as Predictors of IPV Victimisation: The Role of Cultural Context

According to the VSA model, a combination of personal vulnerabilities (e.g., early experiences, personality), environmental stressors (e.g., unemployment, uncertainty), and adaptive processes (e.g., relationship maintenance behaviours, partner support) contributes to variation in relationship quality over time (Karney & Bradbury, 1995). Given the well-established association between relationship quality and the risk of IPV (Ulloa & Hammett, 2015), the VSA model offers a valuable framework for understanding IPV victimisation, particularly under conditions of elevated social stress, such as those seen during the COVID-19 pandemic (Pietromonaco & Overall, 2024). Evidence indicates that IPV increased globally during this period (Peitzmeier et al., 2022). Indeed, the core principle of the VSA model is supported by several studies demonstrating associations between individual vulnerability, pandemic-related stress, and the quality of intimate relationships (Smrdu et al., 2023; Kozakiewicz et al., 2023). Specifically, the COVID-19 pandemic and its associated social distancing measures have been linked to increased relationship conflict (Luetke et al., 2020), likely due to pandemic-related stressors such as job loss and uncertainty, which may exacerbate tensions and reduce couples’ capacity to adapt (Tremblay et al., 2025).

Consistent with the VSA model, a couple’s capacity to adapt to stressful situations is shaped by individual vulnerabilities (Karney & Bradbury, 1995). One important individual vulnerability is attachment style, which refers to the internal working models formed through early relationships with caregivers (Ainsworth & Bowlby, 1991). These models influence how individuals perceive and behave in adult romantic relationships (Hazan & Shaver, 1994). Insecure attachment styles, including anxiety and avoidance, are especially relevant to the study of IPV victimisation. Attachment anxiety is typically characterised by fear of rejection and heightened concerns about the stability of the relationship (Bartholomew & Horowitz, 1991; Hazan & Shaver, 1994). Individuals with anxious attachment tend to be highly sensitive to perceived threats and stress, and while they are often more attuned to their emotions, they may struggle to regulate them (Shaver et al., 2019). In turn, individuals with avoidant attachment tend to value relational independence and often avoid interpersonal closeness and emotional disclosure. They are often less aware of their emotional states and more likely to suppress or mask their emotions (Bartholomew & Horowitz, 1991; Shaver et al., 2019). Because attachment styles affect how individuals identify, process, and regulate their emotions, insecure attachment patterns partly shape the way individuals respond to conflict, making anxious and avoidant individuals particularly vulnerable to IPV victimisation (Feeney & Karantzas, 2017).

Indeed, several meta-analyses and systematic reviews have found that both anxious and avoidant attachment styles are associated with various forms of IPV victimisation (Stefania et al., 2023; Velotti et al., 2018). While most studies report positive associations between insecure attachment and IPV victimisation, the strength and nature of these associations vary depending on the type of violence examined, the target population, and the specific attachment construct measured (Velotti et al., 2018). Meta-analytic evidence, primarily based on cross-sectional data, has found no significant differences in the strength of association between avoidant and anxious attachment styles and IPV victimisation, with both demonstrating moderate effect sizes (Spencer et al., 2021; Stefania et al., 2023). However, longitudinal findings are limited. One exception is a prospective study involving 133 college women in the U.S., which found that attachment anxiety predicted physical IPV victimisation six months later, whereas avoidance did not (Sandberg et al., 2018). This finding suggests that the effects of insecure attachment styles on IPV victimisation may differ over time, with attachment anxiety potentially exerting a sustained impact, while the role of avoidance may be more context-dependent or less stable across time.

Although insecure attachment styles are theorised to increase vulnerability to IPV victimisation, current findings remain mixed. In addition, there is a notable lack of longitudinal research, particularly among general population samples outside the U.S. This gap is especially important in the context of the COVID-19 pandemic, where attachment orientation has emerged as a salient dispositional factor influencing responses to stress (Gottlieb & Schmitt, 2023). Traits associated with insecure attachment, particularly attachment anxiety, appear to have intensified during the pandemic, likely due to increased uncertainty and social isolation (Gruneau Brulin et al., 2022). Importantly, consistent with the VSA model, these personal traits, along with environmental stressors, such as pandemic stress, are likely to influence the adaptive strategies people use in their relationships, which in turn may impact not only the quality of the relationship but also the risk of IPV victimisation.

Moreover, the COVID-19 pandemic fostered an environment marked by stress and uncertainty, which encouraged individuals to engage in strategies aimed at retaining their romantic partners and maintaining their relationships (Degiuli et al., 2023). These relationship maintenance behaviours are known as mate-retention tactics and are adaptive mechanisms that help prevent partner infidelity and defection from the relationship (Buss, 1988). Because mate retention tactics encompass both benefit-provisioning (BP) tactics (i.e., tactics aimed at increasing one’s partner’s self-esteem by providing their partner with benefits) and cost-inflicting (CI) tactics (i.e., tactics aimed at reducing one’s partner’s self-esteem by inflicting cost on the partner), depending on the tactics employed, these may also lead to or exacerbate conflict between partners (Miner et al., 2009). Cross-sectional evidence has shown that the frequency of CI mate-retention strategies is positively associated with abuse (Bhogal & Wallace, 2022), sexual coercion (Lopes et al., 2016), and IPV perpetration (e.g., Kaighobadi et al., 2008). Additionally, CI strategies may emerge from and contribute to relational conflict, thereby perpetuating cycles of violence. This consistent pattern observed between CI strategies and relationship quality helps support these assertions (Altgelt & Meltzer, 2019; Nascimento & Little, 2022), leading some scholars to argue that IPV may, in part, represent an extreme form of mate-retention behaviour (Larsen, 2023). Moreover, research on partner dynamics suggests that people often reciprocate partner behaviour whether positive or negative (Gaines, 1996; Salazar, 2015), implying that one’s own use of CI strategies may be associated not only with IPV perpetration, but also with increased vulnerability to IPV victimisation. This highlights the need to consider individuals’ own mate-retention behaviours, particularly cost-inflicting tactics, as potential contributors to IPV victimisation.

In contrast, BP strategies may reflect healthier dynamics and act as a buffer against IPV. Indeed, research in Iran has found that BP strategies are associated with greater intimacy, maturity, and healthier communication patterns (Ghanbarian et al., 2020). Importantly, both types of mate-retention behaviour have been associated with insecure attachment styles (Barbaro et al., 2021), suggesting that individual vulnerability factors may shape both how people manage relationship threats and their experiences of IPV victimisation. These behaviours may reflect, and potentially reinforce, broader patterns of relational conflict, emotional dysregulation, and reduced partner responsiveness that characterise the relationship, particularly in the context of insecure attachment and elevated relational stress. Taken together, these findings position mate-retention tactics as a natural counterpart to attachment orientation and COVID-19-related stress within the VSA model, representing the adaptive relational processes through which individuals attempt to manage relationship threats, alongside personal vulnerabilities and environmental stressors, respectively. While the pandemic introduced a range of relational strains, including caregiving burden and shifts in the division of labour, mate-retention tactics were of particular interest here given their direct relevance to relationship maintenance and their well-established links to conflict and abuse, discussed further below.

Beyond these individual and relational processes, broader contextual factors may also have shaped IPV risk during this period. Intercountry comparisons have revealed substantial variation in pandemic outcomes, which may have either mitigated or intensified COVID-19-related stressors (Pietromonaco & Overall, 2024). For example, as of April 2022, Brazil had reported over 30 million confirmed cases of COVID-19, ranking third among the world’s most affected countries, with a death rate of 2.18. In comparison, the United Kingdom reported over 21 million cases during the same period, ranking sixth globally, with a lower death rate of 0.79 (Statista, 2023). In Brazil, the federal government initially downplayed the severity of the pandemic, resulting in the absence of a coordinated national response (Ferigato et al., 2020). Similarly, the UK government was criticised for its delayed response, which has been linked to higher mortality rates in comparison to other countries in Europe (Rietveld et al., 2024). These shortcomings suggest that both Brazil and the UK were particularly vulnerable to pandemic-related stressors (Burkova et al., 2021), making them relevant contexts in which to examine the impact of COVID-19 and its associated stressors on rates of IPV victimisation.

Beyond these structural and policy differences, Brazil and the UK also differ in broader cultural orientations that may shape how individuals experience and respond to relational stress. Brazil is typically characterised as a more collectivistic culture, with a stronger emphasis on family interdependence, communal support, and relational obligation (Hofstede, 2001; Santos et al., 2017), whereas the UK is typically characterised as more individualistic, with a comparatively greater emphasis on personal autonomy, independence, and emotional self-reliance (Hofstede, 2001). These differing cultural orientations may shape norms around emotional expression, conflict management, and expectations of closeness within romantic relationships (Lin et al., 2017), and may therefore be relevant to understanding how attachment-related behaviours and relationship maintenance strategies relate to IPV risk across these two contexts. Nonetheless, national-level cultural dimensions such as these represent broad average tendencies rather than measured individual-level values and may not fully capture the heterogeneity within each country; as such, they are considered here as one of several plausible contextual influences, alongside the structural and policy factors outlined above.

Taken together, these structural, policy, and cultural differences underscore the value of examining IPV within both countries as distinct, yet comparable, national contexts. A comprehensive understanding of IPV dynamics, however, requires longitudinal research to examine how stress exposure (e.g., to the COVID-19 pandemic) and relationship behaviours unfold and interact within these contexts over time. The current study addresses this gap by exploring the longitudinal impact of pandemic-related stressors, individual vulnerability, and relationship-related behaviours on psychological, sexual, and physical IPV victimisation in two culturally and structurally distinct contexts: Brazil and the United Kingdom. Specifically, we aimed to achieve the following:

(1) To compare scores of IPV victimisation between Brazil and the UK.
(2) To identify changes in IPV victimisation scores over time.
(3) To determine whether COVID-19-related stressors (i.e., uncertainty, perceived risk, and impact), individual differences (i.e., avoidant and anxious attachment), and relationship-related behaviours (i.e., cost-inflicting and benefit-provisioning mate-retention tactics) predict IPV scores over time and whether these predictors vary by country.

## 2. Method

### 2.1. Participants

Participants were recruited through Qualtrics Panels in Brazil and the United Kingdom. As shown in Table 1, a total of 1416 participants completed the Wave 1 survey (Brazil: *n* = 718; UK: *n* = 698), of whom 353 completed the one-month follow-up survey (Brazil: *n* = 188; UK: *n* = 165). Attrition analyses were conducted to examine whether participants who completed both waves differed from those who only participated at Wave 1 and are reported in the Results section. In Wave 1, Brazilian participants ranged in age from 19 to 79 years (*M* = 41.32, *SD* = 11.74). Most were women (54.9%) and identified as White (59.8%), whereas 40.2% identified as belonging to an ethnic minority group (e.g., Black or mixed-race). Most participants were in an opposite-sex relationship (87.7%), with 12.3% in a same-sex relationship, and the majority had been in their current relationship for more than 5 years (88.5%). In turn, British participants were aged between 31 and 84 (*M* = 51.25, *SD* = 15.22), were mostly men (57.7%), White (95.5%), while a small proportion identified as an ethnic minority (e.g., Black, South Asian, 4.5%). Most participants were in an opposite-sex relationship (92.1%) and in a same-sex relationship (7.9%), and the majority had been in their current relationship for more than 5 years (98.8%). At baseline, Brazilian participants were significantly younger than British participants, *t*(1221.28) = −13.56, *p* < 0.001. The Brazilian sample also included a significantly higher proportion of women than the UK sample (54.9% vs. 42.3%), χ^2^(1, *N* = 1411) = 22.28, *p* < 0.001, φ = 0.13, and a significantly higher proportion of participants identifying as belonging to an ethnic minority group (40.2% vs. 4.5%), χ^2^(1, *N* = 1374) = 237.59, *p* < 0.001, φ = 0.42.

### 2.2. Materials

***The Impact of the COVID-19 Pandemic Questionnaire***, developed for this study, assessed the extent to which individuals were affected by the COVID-19 pandemic, capturing three dimensions: COVID-19 impact, COVID-19 uncertainty, and perceived COVID-19 risk. COVID-19 impact was evaluated by asking participants whether they had experienced any of nine potential pandemic-related consequences, such as becoming unemployed and/or having to care for vulnerable family members. A composite score ranging from 0 to 9 was computed based on the number of endorsed consequences. Because the items represented distinct experiences that collectively formed the index, rather than interchangeable indicators of a single latent construct, internal consistency was not assessed. COVID-19 uncertainty was measured using three items (e.g., *How uncertain do you think your future is as a result of the pandemic?*), each rated on a scale from 0 (*not at all*) to 10 (*extremely uncertain*). A composite score was calculated by averaging the scores of the three items (Cronbach’s alpha: Brazil = 0.84; UK = 0.90). COVID-19 risk was assessed with a single item (*To what extent do you think coronavirus poses a risk to you personally, prior to receiving a coronavirus (COVID-19) vaccination (if you have received one)?*), rated on a scale from 1 (*no risk at all*) to 5 (*major risk*).

**The** ***Relationship Behaviour Rating Scale–Revised*** (RBRS-R; adapted from Beck et al., 2013) is an 18-item scale used to measure IPV. The scale was translated and back-translated into Portuguese by two bilingual researchers to measure IPV in Brazil. The scale measures psychological (e.g., *My partner screamed or yelled at me*), physical (e.g., *My partner tried to choke or strangle me*), and sexual (e.g., *My partner insisted on sex whether I wanted it or not*) IPV victimisation. Participants indicated the frequency of IPV behaviours with statements across 18 items on a 7-point scale, ranging from 1 (*none of the time*) to 7 (*all of the time*). Average scores were calculated separately for the psychological, physical, and sexual IPV subscales, with higher scores indicating greater victimisation. All factors reached acceptable levels of reliability (all Cronbach’s alpha > 0.94 in the UK and > 0.95 in Brazil).

Prior to the main analyses, multi-group confirmatory factor analyses were conducted to evaluate measurement invariance of the RBRS-R scale between Brazil and the UK. Based on the original three-factor structure (psychological, physical, and sexual IPV), configural, metric, and scalar invariance were sequentially tested. The results supported configural invariance (CFI = 0.947, RMSEA = 0.108, SRMR = 0.059), metric invariance (ΔCFI = −0.004, ΔRMSEA = 0.001), and scalar invariance (ΔCFI = −0.001, ΔRMSEA = −0.002), indicating that the IPV scale demonstrated scalar invariance and that scores could be meaningfully compared across countries.

***The Experience in Close Relationships Questionnaire*** (ECR; Wei et al., 2007) was used to measure anxious and avoidant attachment in the UK, which consists of 12 items. In Brazil, a 10-item version adapted to the Brazilian context was used (Natividade et al., 2015). Both versions measured avoidant (e.g., *I try to avoid getting too close to my partner*) and anxious attachment (e.g., *I worry that romantic partners will not care about me as much as I care about them*), with items answered on a seven-point scale (1 = *strongly disagree*; 7 = *strongly agree*). During the validation of the Brazilian version, one anxiety item and one avoidance item from the original short form were removed because they demonstrated poor psychometric performance in the Brazilian sample, resulting in improved model fit and slightly higher internal consistency (Natividade et al., 2015). Thus, although the Brazilian version contains fewer items, it represents the validated adaptation of the same underlying attachment constructs for use in the Brazilian context. In the present study, internal consistency was satisfactory for anxious (Cronbach’s alpha: Brazil = 0.78; UK = 0.79) and avoidant attachment behaviours in both countries (Cronbach’s alpha: Brazil = 0.68; UK = 0.64).

***The Mate Retention Inventory—Short Form*** (MRI-SF; Buss et al., 2008) was used to measure mate-retention tactics by asking participants to rate how often they performed each tactic over the past year. In Brazil, we used the Portuguese version (Lopes et al., 2016). The questionnaire includes 38 items that measure cost-inflicting (e.g., *Did not take your partner to a party where people they might be attracted to would be present*) and benefit-provisioning strategies (e.g., *Displayed affection for your partner*) on a four-point Likert Scale (0 = *never performed this act*; 3 = *often performed this act*). Internal consistency was excellent for cost-inflicting (Cronbach’s alpha: Brazil = 0.91; UK = 0.98) and benefit-provisioning strategies in both countries (Cronbach’s alpha: Brazil = 0.85; UK = 0.93).

### 2.3. Procedure

This study received ethical approval from the Research Ethics Committee at a university in the UK (Reference: 32083-MHR-Dec/2021-35333-2). Data were collected online via Qualtrics, with participants from both countries recruited through Qualtrics Panels, a survey management company. Wave 1 data collection occurred in May 2022, followed by Wave 2 in June 2022. Before completing the survey, participants were screened for eligibility and provided informed consent. The participant information sheet explicitly described the nature of the study to ensure that consent was fully informed. Participants were also informed that they could withdraw from the study at any time without penalty and were free to skip any questions they did not wish to answer. Country-specific support resources and helpline information for individuals affected by intimate partner violence were provided in both the participant information sheet and the debrief form. The same survey questions were used in both waves. Each participant was assigned a random ID, which was later used to link responses across waves. The survey took approximately 30 min to complete.

### 2.4. Data Analysis Strategy

All analyses were conducted using IBM SPSS version 29 and R version 4.6.0 using R Studio. Descriptive statistics were calculated to describe the sample and the main variables of interest. A preliminary correlation analysis was conducted to identify associations between IPV scores at T1 (Wave 1) and T2 (Wave 2), demographic variables (e.g., age, country), COVID-19 stressors at T1, attachment insecurity, and mate-retention strategies at T1 for each type of IPV.

Next, linear mixed models (LMMs) were conducted to examine short-term changes in IPV victimisation across countries, and to investigate the effects of COVID-19 stressors, attachment insecurity, and mate-retention strategies on each type of IPV. Two-way interaction terms between wave and country, and between wave and COVID-19 stressors (i.e., variables expected to fluctuate over a short period of time), were included in the models. Two-way interaction terms between country and each predictor were also included, as we expected these associations to differ between countries. Three-way interaction terms between country, wave, and each COVID-19 stressor were also included. Subject identity was included as a random intercept to account for the non-independence of repeated observations within participants, whereas all predictors and interaction terms were specified as fixed effects. A variance components covariance structure was specified for the random intercept, and a diagonal covariance structure was specified for the repeated residuals. Models were estimated using restricted maximum likelihood (REML), with degrees of freedom for fixed effects estimated using the Satterthwaite approximation. A random intercept was specified for participants, and separate residual variances were estimated for each wave. This likelihood-based approach used all available outcome data and did not require participants to have complete data at both waves; therefore, participants with data from only one wave contributed all available observations to the analyses. This approach assumes that data were missing at random (MAR), conditional on the observed variables included in the models (Rubin, 1976). Under the MAR assumption, the probability of missingness may depend on observed variables but not on unobserved outcome values after conditioning on the observed data (Little, 2021). Because the MAR assumption cannot be verified from the observed data alone, its plausibility was evaluated through an attrition analysis comparing participants who completed both waves with those who only completed Wave 1 on demographic characteristics and baseline study variables. Following the reviewers’ recommendation, the final models were adjusted for age, sex, ethnicity, and relationship length. For transparency, the unadjusted models are reported in the Supplementary Materials (see Supplementary Table S3). Simple slope analyses were conducted to probe significant interaction effects.

Model diagnostics, including histograms, Q-Q plots, and residual-versus-fitted plots, were examined to assess the assumptions of the linear mixed models. Although the IPV outcomes were positively skewed, the diagnostic plots did not indicate departures sufficiently severe to warrant transformations or alternative modelling approaches (please see Supplementary Figures S1–S3).

## 3. Results

### 3.1. Attrition Analysis

Participants who completed both waves (*n* = 353) were compared with those who only completed Wave 1 (*n* = 1063) on demographic characteristics and all baseline study variables (see Supplementary Table S2). Compared with completers, participants who dropped out after Wave 1 were significantly younger, *t*(1407) = 9.08, *p* < 0.001, reported higher levels of psychological, *t*(1413) = −6.79, *p* < 0.001, physical, *t*(1413) = −7.17, *p* < 0.001, and sexual IPV victimisation, *t*(1413) = −6.99, *p* < 0.001, greater baseline COVID-19 uncertainty, *t*(1365) = −4.77, *p* < 0.001, and greater use of both cost-inflicting, *t*(1414) = −7.27, *p* < 0.001, and benefit-provisioning mate-retention strategies, *t*(1414) = −4.94, *p* < 0.001. In contrast, participants who dropped out reported lower levels of anxious attachment, *t*(1414) = 3.64, *p* < 0.001, and avoidant attachment, *t*(1414) = 2.87, *p* = 0.004. No significant differences were observed between completers and non-completers with respect to sex, χ^2^(1, *N* = 1416) = 0.82, *p* = 0.365, country, χ^2^(1, *N* = 1416) = 0.003, *p* = 0.954, ethnicity, χ^2^(1, *N* = 1416) = 0.058, *p* = 0.810, sexual orientation, χ^2^(1, *N* = 1416) = 0.608, *p* = 0.436, baseline COVID-19 risk, *t*(1370) = −1.60, *p* = 0.109, or baseline COVID-19 impact, *t*(1272) = 0.95, *p* = 0.344. These findings indicate that attrition was selective rather than completely random, with participants reporting higher baseline IPV victimisation, greater COVID-19 uncertainty, and greater use of mate-retention behaviours being less likely to complete the follow-up assessment.

Bivariate correlations among all study variables are presented in Supplementary Table S1. Overall, psychological, physical, and sexual IPV were positively intercorrelated both within and across waves. Attachment avoidance and cost-inflicting mate retention strategies showed positive associations with IPV, whereas attachment anxiety and benefit-provisioning strategies demonstrated weaker and more variable associations. These bivariate findings should be interpreted cautiously, as the primary hypotheses were tested using linear mixed-effects models.

### 3.2. LMM Findings

#### 3.2.1. Main Effects

The LMM results indicated that, controlling for age, sex, ethnicity, and relationship length, no main effect of wave/time was observed on any form of IPV. A main effect of country was observed for psychological IPV only, such that Brazilian participants reported higher levels of psychological IPV than UK participants (see Table 2). No main effects were observed for COVID-19-related variables for any type of IPV. Avoidance was negatively associated with both psychological and sexual IPV, but not with physical IPV. In contrast, cost-inflicting mate-retention strategies were positively associated with all types of IPV victimisation, whereas benefit-provisioning strategies were negatively associated with physical and sexual IPV. Full models with degrees of freedom and model fit indicators are provided in the Supplementary Materials (Tables S4.1 and S4.2).

No significant Wave × Country, Wave × COVID-19-related variable, or Country × Wave × COVID-19-related variable interactions were observed for psychological, physical, or sexual IPV. Thus, there was no evidence that changes in IPV across the one-month interval differed by country or according to COVID-19-related uncertainty, perceived risk, or impact.

#### 3.2.2. Two-Way Interactions

Several significant two-way interactions were observed. Specifically, significant interactions between country and attachment avoidance, and between country and cost-inflicting mate retention strategies, were observed for all forms of IPV. In contrast, significant interactions between country and attachment anxiety, and between country and benefit-provisioning mate retention strategies, were observed only for psychological IPV. Simple slopes analyses were conducted to probe these interactions and are reported below.

**Figure 1:**
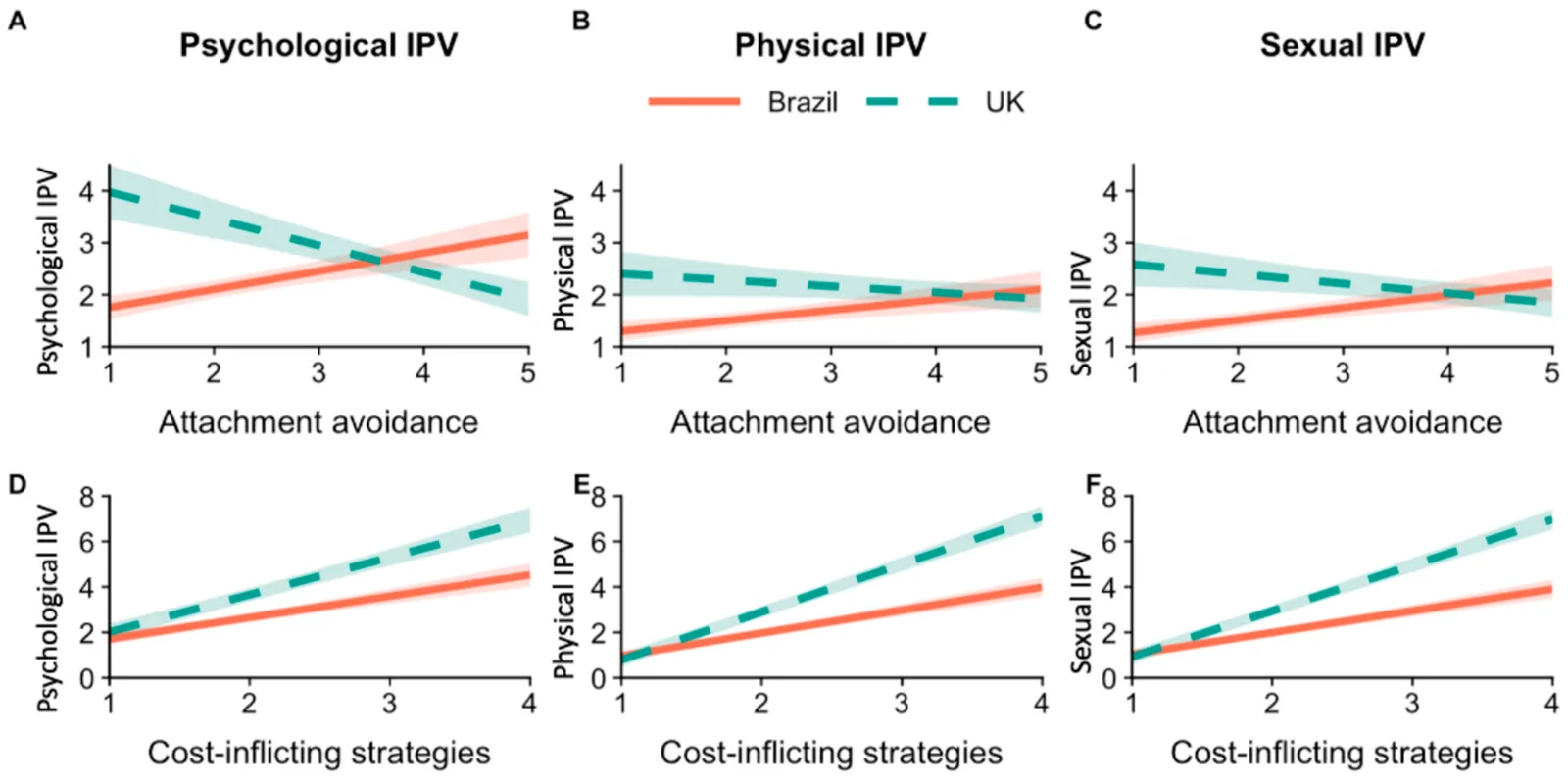
Two-way interactions between attachment and cost-inflicting strategies on psychological (**A**,**D**), physical (**B**,**E**), and sexual (**C**,**F**) IPV.

**Avoidance × country.** Simple slopes analyses showed that attachment avoidance was positively associated with psychological IPV in Brazil, *b* = 0.35, SE = 0.07, *t* = 4.97, *p* < 0.001, and 95% CI [0.21, 0.49], whereas in the UK this association was negative, *b* = −0.51, SE = 0.09, *t* = −5.92, *p* < 0.001, and 95% CI [−0.69, −0.34] (see Figure 1A). The difference between the country-specific slopes was significant, *b* = −0.86, SE = 0.11, *t* = −7.69, *p* < 0.001, and 95% CI [−1.08, −0.64]. Similarly, attachment avoidance was positively associated with physical IPV in Brazil, *b* = 0.20, SE = 0.06, *t* = 3.62, *p* < 0.001, and 95% CI [0.09, 0.31], whereas the association was negative but not statistically significant in the UK, *b* = −0.12, SE = 0.07, *t* = −1.73, *p* = 0.084, and 95% CI [−0.26, 0.02] (see Figure 1B). The difference between the country-specific slopes was also significant, *b* = −0.32, SE = 0.09, *t* = −3.60, *p* < 0.001, and 95% CI [−0.50, −0.15]. In turn, attachment avoidance was positively associated with sexual IPV in Brazil, *b* = 0.24, SE = 0.06, *t* = 4.31, *p* < 0.001, and 95% CI [0.13, 0.35], whereas it was negatively associated with sexual IPV in the UK, *b* = −0.18, SE = 0.07, *t* = −2.67, *p* = 0.008, and 95% CI [−0.32, −0.05] (see Figure 1C). The difference between the country-specific slopes was significant, *b* = −0.42, SE = 0.09, *t* = −4.76, *p* < 0.001, and 95% CI [−0.60, −0.25].

**Cost-inflicting strategies × country.** Simple slopes analyses showed that greater use of cost-inflicting mate retention strategies was positively associated with psychological IPV in both Brazil, *b* = 0.94, SE = 0.11, *t* = 8.77, *p* < 0.001, and 95% CI [0.73, 1.15], and the UK, *b* = 1.64, SE = 0.12, *t* = 13.62, *p* < 0.001, and 95% CI [1.41, 1.88]. The association was significantly stronger in the UK than in Brazil, *b* = 0.70, SE = 0.16, *t* = 4.45, *p* < 0.001, and 95% CI [0.39, 1.02] (see Figure 1D). Greater use of cost-inflicting mate retention strategies was positively associated with physical IPV in both Brazil, *b* = 1.01, SE = 0.09, *t* = 11.82, *p* < 0.001, and 95% CI [0.84, 1.18], and the UK, *b* = 2.10, SE = 0.10, *t* = 22.05, *p* < 0.001, and 95% CI [1.92, 2.29]. The association was significantly stronger in the UK than in Brazil, *b* = 1.09, SE = 0.13, *t* = 8.71, *p* < 0.001, and 95% CI [0.85, 1.34] (see Figure 1E). Greater use of cost-inflicting mate retention strategies was positively associated with sexual IPV in both Brazil, *b* = 0.96, SE = 0.09, *t* = 11.24, *p* < 0.001, and 95% CI [0.79, 1.13], and the UK, *b* = 2.01, SE = 0.10, *t* = 21.13, *p* < 0.001, and 95% CI [1.83, 2.20]. The association was significantly stronger in the UK than in Brazil, *b* = 1.06, SE = 0.13, *t* = 8.41, *p* < 0.001, and 95% CI [0.81, 1.30] (see Figure 1F).

**Figure 2:**
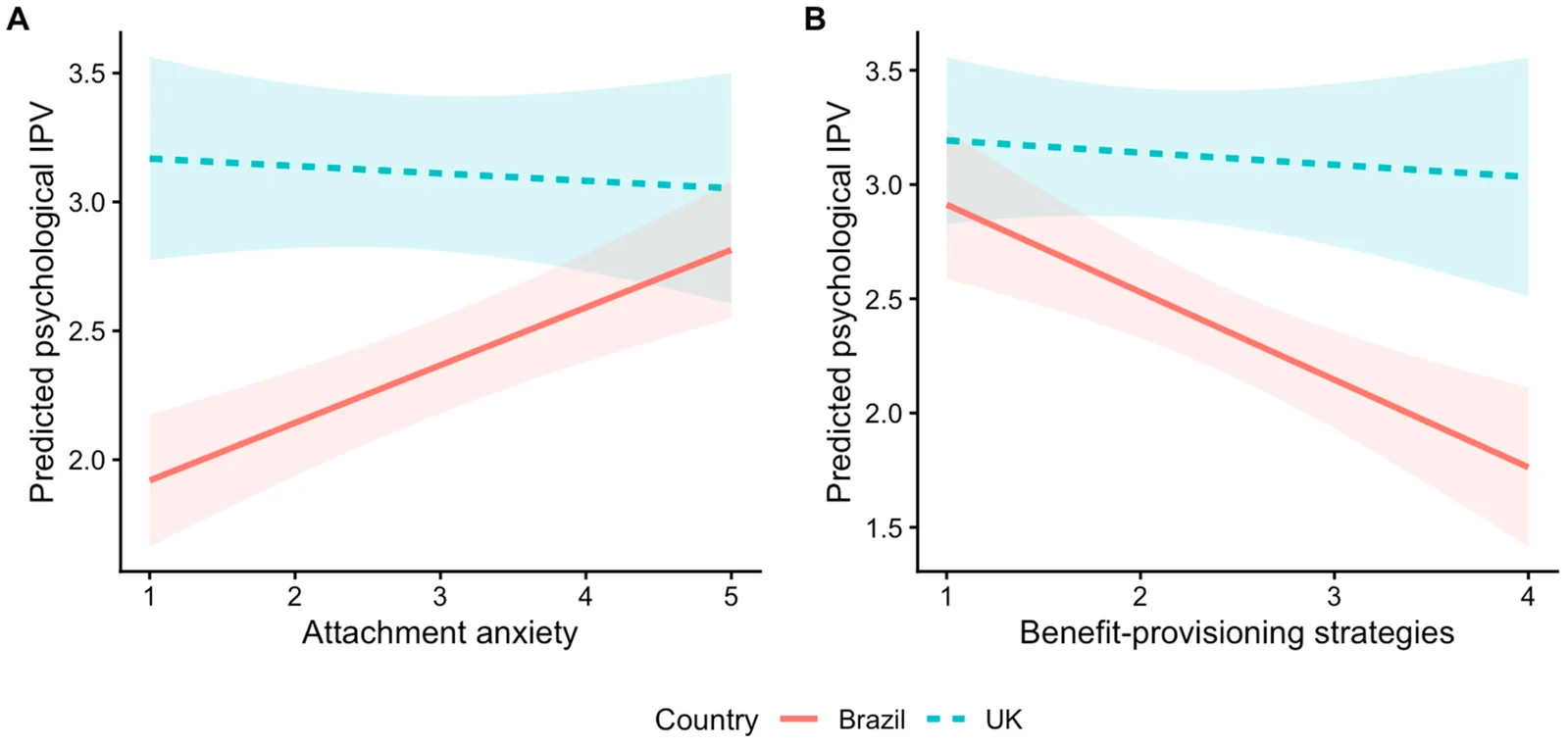
Two-way interactions between country and anxiety (**A**), and benefit-provisioning strategies (**B**) on psychological IPV.

**Attachment anxiety × country.** Simple slopes analyses showed that attachment anxiety was positively associated with psychological IPV in Brazil, *b* = 0.22, SE = 0.05, *t* = 4.769, *p* < 0.001, and 95% CI [0.13, 0.31], whereas the association was non-significant in the UK, *b* = −0.03, SE = 0.07, *t* = −0.38, *p* = 0.70, and 95% CI [−0.17, 0.12]. The difference between the country-specific slopes was significant, *b* = −0.25, SE = 0.09, *t* = −2.85, *p* = 0.004, and 95% CI [−0.42, −0.07] (see Figure 2A).

**Benefit-provisioning × country.** Simple slopes analyses showed that benefit-provisioning mate retention strategies were negatively associated with psychological IPV in Brazil, *b* = − 0.38, SE = 0.09, *t* = − 4.03, *p* < 0.001, and 95% CI [− 0.57, − 0.19], whereas the association was not statistically significant in the UK, *b* = − 0.05, SE = 0.11, *t* = − 0.47, *p* = 0.642, and 95% CI [−0.28, 0.17]. The difference between the country-specific slopes was significant, *b* = 0.33, SE = 0.15, *t* = 2.24, *p* = 0.025, and 95% CI [0.04, 0.62] (see Figure 2B).

## 4. Discussion

The findings of this study are broadly consistent with the Vulnerability–Stress–Adaptation (VSA) model, suggesting that individual vulnerabilities and relationship processes are associated with intimate partner violence (IPV) victimisation over a short period of time. Specifically, the findings highlight the importance of attachment orientations and mate-retention strategies in understanding short-term changes in IPV victimisation, while also revealing meaningful cross-country differences in these associations between Brazil and the United Kingdom, which we interpret considering both cultural and structural contextual factors. The longitudinal design provides insight into short-term changes in IPV victimisation, extending previous cross-sectional research by examining prospective associations across a one-month interval.

### 4.1. Primary Influences on IPV

Attachment styles and mate-retention strategies emerged as significant predictors of IPV victimisation, highlighting how individual dispositions and relationship aspects influence different forms of IPV victimisation independently.

Specifically, avoidant attachment was a key dispositional factor implicated in IPV victimisation. Our findings suggested that, overall, an increase in avoidance was associated with a reduction in IPV rates across psychological and sexual domains. However, we also found that the effect of avoidance on all IPV domains was dependent on country. Specifically, we found that while the association between avoidance and IPV victimisation was negative in the UK, this association was positive in Brazil. Patterns observed in the UK are contrary to much of the existing literature, which associates avoidant attachment styles with an increased risk of IPV (Velotti et al., 2018; Stefania et al., 2023). The divergence of the present results from prior research may be linked to the coping behaviours typically adopted by avoidant individuals, such as avoiding intimacy and emotional closeness (Simpson & Rholes, 2017). These strategies may reduce engagement in emotionally intense interactions that could escalate into IPV in certain cultural contexts, suggesting that avoidance is not always a risk factor. Although direct comparisons between Brazil and the UK are limited, meta-analytic findings have highlighted the role of culture in moderating relationships between attachment avoidance and relationship outcomes (Candel & Turliuc, 2019), with some survey study results highlighting that avoidant behaviours may be particularly likely to produce conflict in collectivistic contexts (e.g., China; Friedman et al., 2010). In certain contexts, perhaps especially those characterised by emotional reservedness, relational indirectness, individualism and autonomy (as in the UK), an increase in avoidant behaviours could serve as a buffer against IPV through emotional disengagement or conflict avoidance. However, these cultural mechanisms were not directly examined in the present study, and this interpretation should therefore be considered speculative. It is also important to note that the UK sample was predominantly White and male. Previous research has consistently shown that men tend to report higher levels of attachment avoidance than women (Del Giudice, 2011). Although sex and ethnicity were included as covariates in the present analyses, differences in the demographic composition of the samples may nevertheless have contributed to the observed cross-country patterns. This possibility warrants further investigation in more demographically comparable samples.

Although no overall association was observed between attachment anxiety and IPV victimisation, a significant interaction between attachment anxiety and country emerged for psychological IPV. Specifically, higher attachment anxiety was associated with greater psychological IPV victimisation in Brazil, whereas the association was non-significant in the UK. Attachment anxiety is characterised by heightened sensitivity to rejection and abandonment, alongside a strong desire for reassurance and relational closeness (Bartholomew & Horowitz, 1991). The interpersonal consequences of these tendencies may depend partly on culturally shaped expectations regarding intimacy, autonomy, and interdependence. In contexts where close relationships are more strongly tied to emotional interdependence and obligations to maintain relational harmony, perceived rejection or withdrawal may be particularly salient to anxiously attached individuals (Lin et al., 2017). This heightened sensitivity could intensify relationship distress and increase vulnerability to psychologically harmful relationship dynamics. Conversely, in more individualistic contexts, where greater autonomy and emotional independence may be more normative (Hofstede, 2001), attachment-related concerns may be expressed or managed differently. However, the present study did not directly assess cultural values, relationship norms, or the behavioural mechanisms underlying these associations. These interpretations therefore remain tentative and require investigation in research that directly measures the relevant cultural and relational processes. It is also possible that cross-national differences in the normative recognition or reporting of psychologically harmful behaviours, rather than differences in the underlying relational dynamics themselves, contribute to this pattern.

In addition to individual vulnerabilities, adaptive relationship behaviours, including CI and BP mate-retention behaviours, were associated with IPV. Firstly, the use of CI strategies was associated with all forms of IPV victimisation. Particularly, our results suggested that individuals who reported using CI strategies also reported higher levels of IPV victimisation. Cost-inflicting behaviours can be viewed as adaptive responses to relational stress and fears of abandonment. However, rather than protecting the relationship, these strategies may be associated with elevated IPV victimisation risk. One possible, though untested, explanation is that engagement in emotionally charged or coercive behaviours could be linked to conflict escalation or mutual hostility. The short one-month interval between waves and the strong temporal stability of both constructs limit our ability to establish clear directionality, and alternative explanations, such as existing victimisation prompting greater use of CI strategies, or a shared underlying pattern of relational conflict that sustains both constructs across waves, remain equally plausible. This is consistent with the literature that reports that bi-directional IPV, especially psychological forms of violence, is a far more commonly observed pattern of relational abuse compared to unidirectional IPV (Machado et al., 2024). The observed relationships between use of CI strategies (e.g., emotionally and psychologically harmful behaviours such as looking through your partner’s belongings, insisting they spend their free time with you, calling to check up on them) and IPV victimisation in this study may be indicative of bi-directional psychological abuse within a partnership. Relatedly, several CI behaviours conceptually resemble controlling or coercive behaviours commonly classified as forms of IPV perpetration. Although the present study measured IPV victimisation rather than perpetration, it is possible that CI strategies as measured here constitute a form of controlling behaviour in their own right. If so, the association between CI strategy use and IPV victimisation may partly reflect conceptual overlap between two related constructs, one’s own controlling behaviour and one’s own experience of victimisation, co-occurring within the same conflictual relational context, rather than a distinct causal or retaliatory process.

Importantly, we also found that the association between CI and all forms of IPV victimisation was stronger in the UK than in Brazil. One possible explanation is that in cultures where autonomy and independence are more strongly valued, such as the UK (Hofstede, 2001; Santos et al., 2017), CI behaviours like snooping or monitoring may be more likely to be interpreted by a partner as a violation of their autonomy or a threat to their independence. Such behaviours may be more likely to provoke defensiveness, emotional withdrawal, or retaliatory aggression, thereby escalating conflict and increasing vulnerability to IPV. Cross-cultural qualitative and quantitative studies have highlighted the role of culture in moderating perceptions of and responses to intrusiveness in romantic relationships, suggesting that in predominantly individualistic contexts (e.g., Australia, United States), people may be more sensitive to intrusion (e.g., perceived invasions of personal space), compared to those living in more collectivistic contexts (e.g., India, Indonesia; Lavy et al., 2009; Sheridan et al., 2017). Conversely, in cultural contexts where interdependence and relational closeness are more strongly emphasised, similar CI behaviours may be perceived as less threatening and may therefore be more likely to be tolerated or normalised within the relationship. If such behaviours are less likely to be experienced as a violation of autonomy, partners may be less inclined to respond with defensiveness, withdrawal, or retaliation, which could help explain the comparatively weaker association between CI and IPV victimisation observed in Brazil.

Evidence also emerged that supportive and warm relationship behaviours, such as spending quality time together, physical and emotional affection, and gift-giving (BP strategies), were associated with lower levels of IPV, particularly sexual and physical forms of victimisation. This finding aligns with the literature indicating that BP strategies, including gift-giving, praise, and affectionate gestures, are associated with greater relationship satisfaction (Ghanbarian et al., 2020; Babaeizad et al., 2022). It is also consistent with Investment Theory (Rusbult et al., 2001), which posits that acts of care and support enhance relationship satisfaction and commitment, thereby fostering a more stable emotional climate in which partners feel valued and respected.

Regarding the role of BP strategies in psychological IPV, a negative association was observed in Brazil only, with no corresponding association found in the UK. This pattern may be interpreted considering experimental work suggesting that, under conditions of heightened interdependence (an attribute more commonly associated with collectivistic cultural contexts; Markus & Kitayama, 1991), gift-giving, particularly material rather than experiential gifts, is associated with greater relationship satisfaction (Liang et al., 2026). It is possible that BP strategies such as gift-giving, expressions of emotional commitment, and prioritisation of quality time are particularly salient to relationship satisfaction in more interdependent relational contexts, which may help explain the protective association observed in the Brazilian sample. In the UK, where relational norms may place comparatively less emphasis on interdependence, these same behaviours may be less central to relationship quality, potentially accounting for the absence of a similar association. Relatedly, individuals higher in interdependent self-construal have been found to be more likely to use gift-giving to express romantic love, compared with those higher in independent self-construal, who may rely more on verbal (spoken or written) expressions of care (Beichen & Murshed, 2015). Again, because we did not test these mechanisms directly, this explanation, although plausible, remains tentative.

After controlling for sex, ethnicity, age, and relationship length, no significant main effect of wave on IPV victimisation emerged, nor was there a significant interaction between wave and country. Given the short, one-month follow-up period, this null finding is perhaps unsurprising, as meaningful change in IPV victimisation may require a longer interval to manifest. It is also worth noting that psychological, physical, and sexual IPV rates were relatively low in both countries even at the outset (Wave 1), which may have further limited the scope for detectable change over such a brief period. This is possibly attributable to data being collected during the later phase of the pandemic (i.e., between May and June 2022), when most restrictions had been lifted and pandemic-related stressors that could exacerbate IPV had been minimised. However, it is worth noting that survey studies using UK, American, and Brazilian samples have identified persistent psychological impacts of COVID-19 in the years following the most severe restrictions (e.g., suicidal ideation, loneliness, self-described mental health as poor; Kohls et al., 2023; Newlove-Delgado et al., 2022; Rosa et al., 2023), suggesting that pandemic-related psychological strain may not fully track with the lifting of formal restrictions. Descriptively, IPV rates in the UK appeared to decline somewhat between waves, while rates in Brazil remained relatively stable; however, as the wave-by-country interaction was not statistically significant, this pattern should be interpreted with caution and not treated as evidence of a genuine cross-national difference in short-term change. Nonetheless, it highlights the value of considering both temporal and cultural factors in future research on IPV dynamics, ideally using longer follow-up periods and larger samples capable of detecting smaller effects.

Similarly, COVID-19-related variables were significantly associated with IPV victimisation in an unadjusted model; however, this association was no longer significant once sex, ethnicity, age, and relationship length were included as covariates. This pattern raises the possibility that the observed association between COVID-19-related uncertainty and IPV victimisation was partly accounted for by demographic factors that may themselves reflect broader structural or socioeconomic vulnerabilities, such as unequal access to financial resources, employment stability, or social support during the pandemic (see Khofi et al., 2025 for a discussion of how such structural vulnerabilities intersect with IPV risk during crises). Rather than indicating that COVID-19-related stressors were unrelated to IPV victimisation, this finding may suggest that demographic characteristics operate as proxies for underlying structural vulnerabilities that were not directly measured in this study. Future research would benefit from directly assessing socioeconomic hardship, access to care, and infrastructural stability during crises, rather than relying on demographic variables as indirect markers of these processes.

### 4.2. Limitations and Future Research Directions

Despite the valuable insights gained from this study, there are several limitations to acknowledge. First, there was a significant dropout rate from Wave 1 (*N* = 1416) to Wave 2 (*N* = 353), with the sample decreasing by 75.1%, which may have introduced an attrition bias and limited the generalisability of the longitudinal results. Indeed, the attrition analyses indicated that participants with higher baseline IPV victimisation and several associated risk factors were less likely to complete Wave 2. However, if follow-up participation was also related to unobserved Wave 2 outcomes, bias may still be present. Accordingly, the longitudinal findings should be interpreted with appropriate caution. Relatedly, the study had a short follow-up period of one month. To explore long-term effects of individual differences on IPV, guidelines suggest implementing more than two waves (Parsons & McCormick, 2024), or more temporal distance between waves (Brandmaier et al., 2024). Importantly, given the short, one-month interval between waves, participants’ Wave 2 responses may have been subject to recall bias, whereby responses were influenced by the memory of, or consistency with, their own Wave 1 reports, rather than reflecting fully independent assessments at each time point.

Additionally, our sample lacked ethnic diversity as most of the participants were White, creating a challenge in the applicability of our findings across different racial and cultural groups. Furthermore, the age and gender distribution were uneven across countries, with British participants being older on average and more likely to be men, while in Brazil the participants included more women and had a broader age range. These disparities may have influenced how relationship dynamics and IPV were experienced and reported across contexts. In this connection, although cross-cultural data were collected, we did not directly measure cultural differences using values, dimensions, or beliefs (e.g., measuring collectivism–individualism; tightness–looseness, etc.); future research should address this disparity using relevant constructs. In terms of recruitment, there are limitations of using online panel services to facilitate data collection (see Eyal et al., 2021); in particular, response rate data (i.e., the proportion of individuals invited who agreed to participate) were not available to the research team, meaning we are unable to assess the extent to which our Wave 1 sample may have differed systematically from the broader pool of individuals invited to participate.

Furthermore, cross-country comparisons may have been confounded by structural differences between Brazil and the UK that were not directly assessed in this study, such as differences in healthcare access, legal protections, and law enforcement responses to IPV. Such factors may independently shape both IPV risk and victims’ willingness or ability to seek help, and future research should directly measure these structural variables to disentangle their contribution from cultural and pandemic-related explanations. This is particularly relevant in the context of the COVID-19 pandemic, during which healthcare systems in Brazil and the UK faced markedly different pressures and capacities (Ferigato et al., 2020; Rietveld et al., 2024), potentially affecting both pandemic-related stress and access to support for IPV victims in ways not captured by the present study. Additionally, the COVID-19 questionnaire was developed for this study and did not undergo formal validation in either country, limiting conclusions about its construct validity and cross-country comparability.

Moreover, the reliance on self-report measures, particularly around a topic which is normatively sanctioned and is generally perceived as not being socially desirable, might have contributed to a self-report bias; however, past research suggests that individuals tend to overestimate their partner’s IPV relative to their own (Follingstad & Edmundson, 2010). Additionally, the use of different versions of the attachment measure across countries represents a limitation of the present study, as measurement invariance of the attachment scale was not assessed. However, it is important to note that both versions have been psychometrically validated within their respective cultural contexts. The Brazilian version was developed from the original 12-item measure, with one anxiety item and one avoidance item removed during the validation process because they demonstrated poor psychometric performance, resulting in improved model fit and internal consistency. Thus, the use of country-specific validated versions reflects an effort to employ culturally appropriate measures of the same underlying attachment constructs, consistent with calls for more culturally emic approaches that prioritise culturally relevant measurement while maintaining conceptual equivalence (Boehnke, 2022). Another important limitation was the low internal consistency of the attachment avoidance factor in both countries, indicating that findings involving attachment avoidance should be interpreted with caution. Therefore, these findings would benefit from replication using measures with stronger reliability.

Overall, future research should aim to recruit a more representative sample across age, gender, and ethnicity, to better capture the complexities and intersectionality of IPV experiences. Additionally, subsequent research can adopt a mixed-method approach to help provide a more comprehensive understanding of the underpinnings of IPV.

## 5. Conclusions and Prevention Implications

In conclusion, the findings of this study were in accordance with the VSA model and indicate that attachment orientations and mate-retention strategies were associated with IPV victimisation and that several of these associations differed between Brazil and the UK. IPV victimisation did not change significantly across the one-month interval, and the adjusted models provided no evidence that COVID-19-related variables were associated with IPV victimisation. This study has important implications for intervention and rehabilitation programmes. It emphasises the need for IPV prevention and recovery programmes to address not just individual risk factors but also consider broader environmental factors and promote positive adaptive relationship strategies. Additionally, this study highlights the need for more culturally sensitive interventions to account for the nuances in how different cultures display and interpret attachment behaviours and mate-retention adaptive strategies, especially during stressful periods like a global crisis.

## Figures and Tables

**Table 1:** Sample demographics and means (standard deviations) of IPV by country and wave.

Wave	Country	*N*	Age (*M*, *SD*)	Percentage				*M*(*SD*)		
				Man	Woman	White	Ethnic Minority	Psychological IPV	Physical	Sexual IPV
T1	Brazil	714	41.32, 11.73	45.1%	54.9%	59.4%	40.2%	1.90 (1.29)	1.36 (1.00)	1.39 (1.00)
T1	UK	698	51.25, 15.22	57.7%	42.3%	95.5%	4.5%	2.75 (2.25)	2.31 (2.28)	2.31 (2.26)
T2	Brazil	188	44.13, 12.30	44.9%	53.1%	62.4%	37.6%	1.93 (1.39)	1.42 (1.12)	1.45 (1.14)
T2	UK	165	60.48, 11.93	53%	47%	93.5%	6.5%	1.59 (1.16)	1.14 (0.70)	1.13 (0.70)

*Note.* N represents the total number of participants per category. Percentages for gender and ethnicity are based on available data. *M* indicates mean, and *SD* indicates standard deviation for age and each IPV type (psychological, physical, and sexual). Baseline demographic comparisons indicated that Brazilian participants were significantly younger than British participants, *t*(1221.28) = −13.56, *p* < 0.001. The Brazilian sample also included a higher proportion of women (54.9% vs. 42.3%), χ^2^(1, *N* = 1411) = 22.28, *p* < 0.001, φ = 0.13, and participants identifying as belonging to an ethnic minority group (40.2% vs. 4.5%), χ^2^(1, *N* = 1374) = 237.59, *p* < 0.001, φ = 0.42.

**Table 2:** Linear mixed-effects models predicting IPV.

	Psychological IPV			Physical IPV			Sexual IPV		
Predictor	*B* (SE)	95% CI	*p*	*B* (SE)	95% CI	*p*	*B* (SE)	95% CI	*p*
Intercept	1.774 (0.689)	[0.421, 3.126]	0.010	−0.254 (0.551)	[−1.336, 0.827]	0.644	0.046 (0.550)	[−1.033, 1.125]	0.933
Wave 1 vs. Wave 2	−0.621 (0.336)	[−1.281, 0.040]	0.065	−0.252 (0.301)	[−0.844, 0.340]	0.403	−0.391 (0.297)	[−0.974, 0.193]	0.189
Brazil vs. UK	**−2.063** (0.674)	[−3.386, −0.740]	0.002	0.782 (0.552)	[−0.302, 1.865]	0.157	0.399 (0.550)	[−0.680, 1.479]	0.468
COVID-19 risk	0.031 (0.092)	[−0.151, 0.212]	0.742	0.054 (0.080)	[−0.103, 0.211]	0.499	0.032 (0.079)	[−0.124, 0.187]	0.691
COVID-19 uncertainty	−0.007 (0.017)	[−0.041, 0.027]	0.691	0.007 (0.015)	[−0.022, 0.036]	0.629	0.010 (0.015)	[−0.018, 0.039]	0.479
COVID-19 impact	−0.007 (0.092)	[−0.187, 0.173]	0.939	−0.039 (0.081)	[−0.198, 0.120]	0.630	−0.026 (0.080)	[−0.183, 0.131]	0.747
Attachment anxiety	−0.029 (0.075)	[−0.176, 0.119]	0.702	0.052 (0.059)	[−0.065, 0.169]	0.381	0.098 (0.059)	[−0.018, 0.215]	0.098
Attachment avoidance	**−0.515** (0.087)	[−0.685, −0.344]	<0.001	−0.120 (0.069)	[−0.256, 0.016]	0.083	**−0.185** (0.069)	[−0.321, −0.049]	0.008
Cost-inflicting mate retention	**1.644** (0.121)	[1.408, 1.881]	<0.001	**2.103** (0.095)	[1.916, 2.290]	<0.001	**2.015** (0.095)	[1.828, 2.202]	<0.001
Benefit-provisioning mate retention	−0.053 (0.114)	[−0.277, 0.171]	0.641	**−0.280** (0.091)	[−0.458, −0.102]	0.002	**−0.236** (0.091)	[−0.414, −0.058]	0.009
Country × COVID-19 risk	−0.001 (0.121)	[−0.237, 0.236]	0.996	−0.081 (0.105)	[−0.286, 0.125]	0.442	−0.045 (0.104)	[−0.249, 0.158]	0.662
Country × COVID-19 uncertainty	0.022 (0.022)	[−0.020, 0.065]	0.303	0.006 (0.019)	[−0.031, 0.042]	0.754	0.002 (0.018)	[−0.034, 0.038]	0.909
Country × COVID-19 impact	−0.040 (0.107)	[−0.251, 0.170]	0.706	−0.043 (0.094)	[−0.228, 0.142]	0.646	−0.050 (0.093)	[−0.233, 0.133]	0.591
Country × Attachment anxiety	**0.252** (0.088)	[0.079, 0.426]	0.004	−0.054 (0.070)	[−0.192, 0.084]	0.443	−0.090 (0.070)	[−0.228, 0.048]	0.200
Country × Attachment avoidance	**0.863** (0.112)	[0.644, 1.083]	<0.001	**0.321** (0.089)	[0.146, 0.497]	<0.001	**0.425** (0.089)	[0.250, 0.600]	<0.001
Country × Cost-inflicting mate retention	**−0.705** (0.158)	[−1.015, −0.394]	<0.001	**−1.095** (0.126)	[−1.341, −0.849]	<0.001	**−1.057** (0.125)	[−1.303, −0.810]	<0.001
Country × Benefit-provisioning mate retention	**−0.330** (0.147)	[−0.618, −0.042]	0.025	0.022 (0.117)	[−0.207, 0.251]	0.850	0.043 (0.117)	[−0.186, 0.272]	0.713
Wave × Country	0.387 (0.466)	[−0.529, 1.303]	0.407	0.109 (0.418)	[−0.713, 0.930]	0.795	0.267 (0.412)	[−0.543, 1.076]	0.518
Wave × COVID-19 risk	0.063 (0.096)	[−0.126, 0.252]	0.515	0.050 (0.086)	[−0.119, 0.218]	0.564	0.084 (0.085)	[−0.083, 0.250]	0.324
Wave × COVID-19 uncertainty	0.028 (0.018)	[−0.008, 0.063]	0.132	0.017 (0.016)	[−0.014, 0.048]	0.287	0.020 (0.016)	[−0.011, 0.051]	0.207
Wave × COVID-19 impact	0.117 (0.100)	[−0.079, 0.314]	0.242	−0.040 (0.088)	[−0.213, 0.134]	0.655	−0.054 (0.087)	[−0.225, 0.118]	0.538
Wave × Country × COVID-19 risk	−0.027 (0.128)	[−0.278, 0.224]	0.832	−0.025 (0.114)	[−0.248, 0.199]	0.828	−0.070 (0.112)	[−0.291, 0.150]	0.531
Wave × Country × COVID-19 uncertainty	−0.034 (0.023)	[−0.079, 0.010]	0.133	−0.022 (0.020)	[−0.061, 0.017]	0.270	−0.023 (0.020)	[−0.062, 0.016]	0.246
Wave × Country × COVID-19 impact	−0.019 (0.117)	[−0.248, 0.211]	0.874	0.075 (0.104)	[−0.129, 0.279]	0.470	0.092 (0.102)	[−0.109, 0.293]	0.370
Age	0.001 (0.003)	[−0.005, 0.008]	0.667	−0.004 (0.002)	[−0.009, 0.001]	0.117	**−0.005** (0.002)	[−0.010, −0.000]	0.040
Female vs. male	−0.097 (0.076)	[−0.246, 0.051]	0.200	**−0.237** (0.058)	[−0.351, −0.124]	<0.001	**−0.157** (0.058)	[−0.270, −0.043]	0.007
Ethnic minority vs. White	−0.047 (0.085)	[−0.214, 0.120]	0.579	−0.058 (0.064)	[−0.185, 0.068]	0.365	−0.069 (0.065)	[−0.196, 0.058]	0.289
Relationship length: 6–12 months	−0.006 (0.459)	[−0.906, 0.894]	0.989	−0.012 (0.359)	[−0.716, 0.692]	0.972	−0.041 (0.359)	[−0.745, 0.662]	0.909
Relationship length: 1–2 years	0.370 (0.448)	[−0.509, 1.250]	0.409	0.089 (0.351)	[−0.599, 0.776]	0.801	−0.019 (0.350)	[−0.707, 0.668]	0.956
Relationship length: 2–3 years	0.063 (0.427)	[−0.774, 0.900]	0.882	0.008 (0.334)	[−0.648, 0.663]	0.982	−0.285 (0.334)	[−0.939, 0.370]	0.393
Relationship length: 3–5 years	0.193 (0.410)	[−0.610, 0.997]	0.637	−0.049 (0.321)	[−0.679, 0.581]	0.879	−0.182 (0.321)	[−0.811, 0.448]	0.571
Relationship length: More than 5 years	0.206 (0.392)	[−0.563, 0.975]	0.599	−0.088 (0.308)	[−0.692, 0.516]	0.775	−0.214 (0.308)	[−0.817, 0.389]	0.486
**Random Effects**	Psychological IPV			Physical IPV			Sexual IPV		
Random intercept variance	0.667			0.156			0.194		
Residual variance	0.674			0.671			0.631		
ICC	0.83			0.82			0.82		

Note. B = unstandardised coefficient; SE = standard error; CI = 95% confidence interval. Models adjusted for age, sex, ethnicity, and relationship length. Wave 2, the UK, male sex, White ethnicity, and relationship length below six months were the reference categories. Models were estimated using restricted maximum likelihood with Satterthwaite degrees of freedom and included a random intercept for participant. Random intercept and residual variance estimates are reported from the final adjusted models. ICC values were obtained from unconditional (intercept-only) models. Significant coefficients are highlighted in bold.

## Data Availability

The full data can be obtained by contacting the corresponding author at nascimento.brunads@gmail.com.

## Supplementary Materials

The following supporting information can be downloaded at https://www.mdpi.com/article/10.3390/bs16081388/s1. Figure S1: Normal Q-Q plots of residuals for Psychological, Physical, and Sexual IPV; Figure S2: Residuals plotted against predicted values for adjusted linear mixed-effects models predicting different forms of IPV; Figure S3: Distribution of residuals for the different IPV models; Table S1: Correlations between IPV types and relationship variables, individual differences, and demographic variables; Table S2: Fixed and Random Effects from LMM Predicting IPV Types (Psychological, Physical, and Sexual); Table S3: Attrition Analysis; Table S4.1: Full Results from the Linear Mixed-Effects Models Predicting Psychological, Physical, and Sexual Intimate Partner Violence; Table S4.2: Model Information.
