# Supplementary material for: Attachment, Mate-Retention Strategies, and Intimate Partner Violence Victimisation: A Two-Wave Study in Brazil and the United Kingdom
Source: Behav Sci (Basel). 2026 Aug 12;16(8):1388. doi: 10.3390/bs16081388 (PMC13509817; doi:10.3390/bs16081388)
Supplement: Supplementary file 1 [file behavsci-16-01388-s001.zip › behavsci-4372460-supplementary.pdf]

Table S1

Correlations between IPV types and relationship variables, individual differences, and demographic variables

Variable	1	2	3	4	5	6	7	8	9	10	11	12	13	14	15	16
1. Psychological IPV T1	—															
2. Physical IPV T1	.871**	—														
3. Sexual IPV T1	.863**	.977**	—													
4. Psychological IPV T2	.669**	.322**	.329**	—												
5. Physical IPV T2	.352**	.392**	.397**	.720**	—											
6. Sexual IPV T2	.322**	.343**	.395**	.698**	.964**	—										
7. COVID risk T1	.161**	.152**	.158**	.086	.064	.065	—									
8. COVID uncertainty T1	.457**	.455**	.467**	.255**	.280**	.277**	.245**	—								
9. COVID impact T1	-.101**	-.207**	-.197**	.082	-.044	-.040	.089**	.062*	—							
10. Own CI T1	.699**	.778**	.777**	.375**	.409**	.412**	.181**	.499**	-.135**	—						
11. Own BP T1	.294**	.362**	.363**	.090	.131*	.135*	.189**	.244**	.019	.628**	—					
12. Anxiety T1	-.087**	-.155**	-.150**	.071	.053	.071	-.182**	-.084**	.014	-.174**	-.241**	—				
13. Avoidance T1	.060*	.080**	.074**	-.010	.037	.026	-.180**	-.106**	-.145**	-.119**	-.378**	.500**	—			
14. UK	.230**	.267**	.260**	-.129*	-.151**	-.168**	-.131**	-.012	-.190**	.023	-.261**	.313**	.788**	—		
15. Ethnic minority	-.121**	-.133**	-.128**	.209**	.223**	.188**	.068*	.043	.096**	.001	.127**	-.146**	-.333**	-.416**	—	
16. Woman	-.221**	-.299**	-.278**	.008	-.046	-.005	-.031	-.048	.207**	-.260**	-.247**	.056*	-.071**	-.126**	.099**	—
17. Relationship length	-.132**	-.177**	-.180**	-.119*	-.250**	-.251**	.017	-.159**	.059*	-.209**	-.111**	.062*	.072**	.045	-.053	.119**

Note. * p < .05, ** p < .01, *** p < .001.

Table S2

Fixed and Random Effects from LMM Predicting IPV Types (Psychological, Physical, and Sexual)

Predictor	Psychological IPV		Physical IPV		Sexual IPV	
	Estimate (SE)	95% CI	Estimate (SE)	95% CI	Estimate (SE)	95% CI
Intercept	2.28 (.51)***	[1.28, 3.28]	-.33 (.41)	[-1.13, .48]	-.21 (.42)	[-1.02, .61]
Fixed effects						
Wave 1	-.92 (.33)**	[-1.56, -.28]	-.63 (.29)*	[-1.19, -.06]	-.76 (.29)**	[-1.33, -.20]
Country Brazil	-2.50 (.65)***	[-3.77, -1.23]	.28 (.53)	[-.75, 1.32]	.01 (.53)	[-1.03, 1.05]
COVID Risk	.05 (.09)	[-.13, .22]	.05 (.07)	[-.09, .19]	.03 (.07)	[-.12, .17]
COVID Uncertainty	-.02 (.02)	[-.06, .01]	-.01 (.01)	[-.03, .02]	-.00 (.01)	[-.03, .02]
COVID Impact	-.01 (.09)	[-.18, .17]	-.05 (.08)	[-.19, .10]	-.03 (.08)	[-.17, .12]
Anxious	-.05 (.07)	[-.19, .09]	.01 (.06)	[-.11, .12]	.05 (.06)	[-.07, .16]
Avoidant	-.52 (.09)***	[-.68, -.35]	-.11 (.07)	[-.25, .03]	-.16 (.07)*	[-.29, -.02]
CI strategies	1.51 (.11)***	[1.29, 1.74]	2.00 (.09)***	[1.82, 2.18]	1.93 (.09)***	[1.74, 2.11]
BP strategies	-.02 (.11)	[-.24, .20]	-.25 (.09)**	[-.43, -.07]	-.23 (.09)*	[-.40, -.05]
Wave × Country	.73 (.46)	[-.17, 1.63]	.48 (.40)	[-.32, 1.27]	.60 (.40)	[-.20, 1.39]
Wave × COVID Risk	.08 (.09)	[-.10, .27]	.09 (.08)	[-.07, .25]	.12 (.08)	[-.04, .28]
Wave × COVID Uncertainty	.06 (.02)**	[.02, .09]	.05 (.02)**	[.02, .08]	.05 (.02)***	[.02, .08]
Wave × COVID Impact	.07 (.10)	[-.12, .27]	-.08 (.09)	[-.25, .09]	-.09 (.09)	[-.26, .07]
Country × COVID Risk	-.04 (.12)	[-.27, .18]	-.10 (.10)	[-.29, .09]	-.06 (.10)	[-.26, .13]
Country × COVID Uncertainty	.04 (.02)*	[.00, .08]	.02 (.02)	[-.01, .05]	.02 (.02)	[-.02, .05]
Country × COVID Impact	-.01 (.10)	[-.22, .19]	-.05 (.09)	[-.22, .13]	-.06 (.09)	[-.23, .12]
Country × Anxious	.27 (.09)**	[.10, .44]	-.03 (.07)	[-.17, .11]	-.06 (.07)	[-.20, .08]
Country × Avoidant	.91 (.11)***	[.69, 1.12]	.37 (.09)***	[.20, .55]	.44 (.09)***	[.26, .62]
Country × CI strategies	-.62 (.16)***	[-.93, -.32]	-1.00 (.13)***	[-1.25, -.75]	-.94 (.13)***	[-1.19, -.69]

Country × BP strategies	-.33 (.15)*	[-.61, -.04]	.06 (.12)	[-.17, .29]	.07 (.12)	[-.16, .31]
Wave × Country × COVID Risk	-.03 (.13)	[-.27, .22]	-.05 (.11)	[-.27, .16]	-.09 (.11)	[-.31, .12]
Wave × Country × COVID Uncertainty	-.07 (.02)**	[-.11, -.02]	-.05 (.02)**	[-.09, -.02]	-.06 (.02)**	[-.09, -.02]
Wave × Country × COVID Impact	-.01 (.12)	[-.24, .22]	.11 (.10)	[-.08, .31]	.13 (.10)	[-.06, .33]
Random effects						
Intercept	.57		.06		.10	
Residual variance (Wave 1)	.85		.92		.86	
Residual variance (Wave 2)	.70		.70		.69	
ICC	.83		.82		.82	

Note. Values are unstandardised regression coefficients (*b*) with standard errors in parentheses and 95% confidence intervals. ICC values were calculated from unconditional (intercept-only) models. Random-effect variance estimates are from the final fitted models. *p* < .05. p < .01. *p* < .001.

Table S3
Attrition Analysis

Variable	Group	N	Mean(S.D.) / n (%)
IPV_PSYCH	completers	353	1.72 (1.25)
	drop-outs	1062	2.48 (1.97)
IPV_PYSH	completers	353	1.22 (0.83)
	drop-outs	1062	2.00 (1.97)
IPV_SV	completers	353	1.25 (0.87)
	drop-outs	1062	2.00 (1.94)
COVIDrisk	completers	343	3.41 (1.11)
	drop-outs	1029	3.52 (1.15)
COVID_uncert	completers	345	12.73 (6.92)
	drop-outs	1022	14.87 (7.30)
COVID_impact	completers	301	1.93 (1.33)
	drop-outs	973	1.85 (1.34)
OwnCI	completers	353	1.42 (0.51)
	drop-outs	1063	1.73 (0.76)
OwnBP	completers	353	2.33 (0.65)
	drop-outs	1063	2.52 (0.65)
Anxious	completers	353	3.10 (1.04)
	drop-outs	1063	2.87 (1.00)
Avoidant	completers	353	2.91 (1.27)
	drop-outs	1063	2.70 (1.15)
Age	completers	353	51.77 (14.61)
	drop-outs	1056	43.99 (13.72)
Sex	completers		Male: 171 (48.6%), Female: 180 (51.1%)
	drop-outs		Male: 548 (51.6%), Female: 512 (48.2%)
Ethnicity	completers		White: 261 (74.8%), Minority: 80 (22.9%)
	drop-outs		White: 782 (73.6%), Minority: 251 (23.6%)
Country	completers		Brazil: 188 (53.3%), UK: 165 (46.7%)
	drop-outs		Brazil: 570 (53.6%), UK: 493 (46.4%)
orientation	completers		Heterosexual: 315 (89.2%), LGBTG+: 36 (10.2%)
	drop-outs		Heterosexual: 967 (91.0%), LGBTQ+: 92 (8.7%)

Table S4.1
Full Results from the Linear Mixed-Effects Models Predicting Psychological, Physical, and Sexual Intimate Partner Violence

Outcome	Predictor	B	SE	95% CI	df	*t*	*p*
Psychological IPV	Intercept	1.774	0.689	[0.421, 3.126]	1367.0	2.57	.010
	Wave 1 vs. Wave 2	-0.621	0.336	[-1.281, 0.040]	454.9	-1.85	.065
	Brazil vs. United Kingdom	-2.063	0.674	[-3.386, -0.740]	1196.5	-3.06	.002
	COVID-19 risk	0.031	0.092	[-0.151, 0.212]	627.3	0.33	.742
	COVID-19 uncertainty	-0.007	0.017	[-0.041, 0.027]	699.4	-0.40	.691
	COVID-19 impact	-0.007	0.092	[-0.187, 0.173]	481.7	-0.08	.939
	Attachment anxiety	-0.029	0.075	[-0.176, 0.119]	1366.6	-0.38	.702
	Attachment avoidance	-0.515	0.087	[-0.685, -0.344]	1361.2	-5.93	< .001
	Cost-inflicting mate-retention tactics	1.644	0.121	[1.408, 1.881]	1359.9	13.64	< .001
	Benefit-provisioning mate-retention tactics	-0.053	0.114	[-0.277, 0.171]	1365.0	-0.47	.641
	Age	0.001	0.003	[-0.005, 0.008]	1074.8	0.43	.667
	Female vs. male	-0.097	0.076	[-0.246, 0.051]	1083.9	-1.28	.200
	Minority ethnicity vs. White ethnicity	-0.047	0.085	[-0.214, 0.120]	1032.8	-0.55	.579
	Relationship length: 6–12 months vs. < 6 months	-0.006	0.459	[-0.906, 0.894]	1229.3	-0.01	.989
	Relationship length: 1–2 years vs. < 6 months	0.370	0.448	[-0.509, 1.250]	1227.2	0.83	.409
	Relationship length: 2–3 years vs. < 6 months	0.063	0.427	[-0.774, 0.900]	1230.4	0.15	.882
	Relationship length: 3–5 years vs. < 6 months	0.193	0.410	[-0.610, 0.997]	1241.5	0.47	.637
	Relationship length: > 5 years vs. < 6 months	0.206	0.392	[-0.563, 0.975]	1248.4	0.53	.599

Outcome	Predictor	B	SE	95% CI	df	*t*	*p*
	Wave 1 × Brazil	0.387	0.466	[-0.529, 1.303]	450.8	0.83	.407
	Wave 1 × COVID-19 risk	0.063	0.096	[-0.126, 0.252]	464.2	0.65	.515
	Brazil × COVID-19 risk	-0.001	0.121	[-0.237, 0.236]	595.9	-0.00	.996
	Wave 1 × COVID-19 uncertainty	0.028	0.018	[-0.008, 0.063]	588.2	1.51	.132
	Brazil × COVID-19 uncertainty	0.022	0.022	[-0.020, 0.065]	644.9	1.03	.303
	Wave 1 × COVID-19 impact	0.117	0.100	[-0.079, 0.314]	507.9	1.17	.242
	Brazil × COVID-19 impact	-0.040	0.107	[-0.251, 0.170]	494.1	-0.38	.706
	Brazil × attachment anxiety	0.252	0.088	[0.079, 0.426]	1367.6	2.85	.004
	Brazil × attachment avoidance	0.863	0.112	[0.644, 1.083]	1363.5	7.71	< .001
	Brazil × cost-inflicting tactics	-0.705	0.158	[-1.015, -0.394]	1367.1	-4.45	< .001
	Brazil × benefit-provisioning tactics	-0.330	0.147	[-0.618, -0.042]	1365.7	-2.25	.025
	Wave 1 × Brazil × COVID-19 risk	-0.027	0.128	[-0.278, 0.224]	473.3	-0.21	.832
	Wave 1 × Brazil × COVID-19 uncertainty	-0.034	0.023	[-0.079, 0.010]	524.4	-1.51	.133
	Wave 1 × Brazil × COVID-19 impact	-0.019	0.117	[-0.248, 0.211]	490.1	-0.16	.874
Physical IPV	Intercept	-0.254	0.551	[-1.336, 0.827]	1323.3	-0.46	.644
	Wave 1 vs. Wave 2	-0.252	0.301	[-0.844, 0.340]	515.8	-0.84	.403
	Brazil vs. United Kingdom	0.782	0.552	[-0.302, 1.865]	1367.8	1.42	.157
	COVID-19 risk	0.054	0.080	[-0.103, 0.211]	948.8	0.68	.499
	COVID-19 uncertainty	0.007	0.015	[-0.022, 0.036]	1040.9	0.48	.629
	COVID-19 impact	-0.039	0.081	[-0.198, 0.120]	814.1	-0.48	.630
	Attachment anxiety	0.052	0.059	[-0.065, 0.169]	1201.9	0.88	.381

Outcome	Predictor	B	SE	95% CI	df	*t*	*p*
	Attachment avoidance	-0.120	0.069	[-0.256, 0.016]	1235.4	-1.73	.083
	Cost-inflicting mate-retention tactics	2.103	0.095	[1.916, 2.290]	1215.7	22.07	< .001
	Benefit-provisioning mate-retention tactics	-0.280	0.091	[-0.458, -0.102]	1215.5	-3.08	.002
	Age	-0.004	0.002	[-0.009, 0.001]	737.9	-1.57	.117
	Female vs. male	-0.237	0.058	[-0.351, -0.124]	749.5	-4.12	< .001
	Minority ethnicity vs. White ethnicity	-0.058	0.064	[-0.185, 0.068]	692.6	-0.91	.365
	Relationship length: 6–12 months vs. < 6 months	-0.012	0.359	[-0.716, 0.692]	1269.0	-0.03	.972
	Relationship length: 1–2 years vs. < 6 months	0.089	0.351	[-0.599, 0.776]	1262.4	0.25	.801
	Relationship length: 2–3 years vs. < 6 months	0.008	0.334	[-0.648, 0.663]	1273.2	0.02	.982
	Relationship length: 3–5 years vs. < 6 months	-0.049	0.321	[-0.679, 0.581]	1301.5	-0.15	.879
	Relationship length: > 5 years vs. < 6 months	-0.088	0.308	[-0.692, 0.516]	1316.2	-0.29	.775
	Wave 1 × Brazil	0.109	0.418	[-0.713, 0.930]	519.6	0.26	.795
	Wave 1 × COVID-19 risk	0.050	0.086	[-0.119, 0.218]	580.1	0.58	.564
	Brazil × COVID-19 risk	-0.081	0.105	[-0.286, 0.125]	926.1	-0.77	.442
	Wave 1 × COVID-19 uncertainty	0.017	0.016	[-0.014, 0.048]	763.6	1.07	.287
	Brazil × COVID-19 uncertainty	0.006	0.019	[-0.031, 0.042]	973.8	0.31	.754
	Wave 1 × COVID-19 impact	-0.040	0.088	[-0.213, 0.134]	714.1	-0.45	.655
	Brazil × COVID-19 impact	-0.043	0.094	[-0.228, 0.142]	824.5	-0.46	.646
	Brazil × attachment anxiety	-0.054	0.070	[-0.192, 0.084]	1247.0	-0.77	.443
	Brazil × attachment avoidance	0.321	0.089	[0.146, 0.497]	1228.6	3.60	< .001
	Brazil × cost-inflicting tactics	-1.095	0.126	[-1.341, -0.849]	1236.9	-8.72	< .001

Outcome	Predictor	B	SE	95% CI	df	*t*	*p*
	Brazil × benefit-provisioning tactics	0.022	0.117	[-0.207, 0.251]	1209.5	0.19	.850
	Wave 1 × Brazil × COVID-19 risk	-0.025	0.114	[-0.248, 0.199]	602.5	-0.22	.828
	Wave 1 × Brazil × COVID-19 uncertainty	-0.022	0.020	[-0.061, 0.017]	672.0	-1.10	.270
	Wave 1 × Brazil × COVID-19 impact	0.075	0.104	[-0.129, 0.279]	668.3	0.72	.470
Sexual IPV	Intercept	0.046	0.550	[-1.033, 1.125]	1346.2	0.08	.933
	Wave 1 vs. Wave 2	-0.391	0.297	[-0.974, 0.193]	540.2	-1.32	.189
	Brazil vs. United Kingdom	0.399	0.550	[-0.680, 1.479]	1357.1	0.73	.468
	COVID-19 risk	0.032	0.079	[-0.124, 0.187]	902.5	0.40	.691
	COVID-19 uncertainty	0.010	0.015	[-0.018, 0.039]	995.1	0.71	.479
	COVID-19 impact	-0.026	0.080	[-0.183, 0.131]	760.2	-0.32	.747
	Attachment anxiety	0.098	0.059	[-0.018, 0.215]	1262.4	1.65	.098
	Attachment avoidance	-0.185	0.069	[-0.321, -0.049]	1295.4	-2.68	.008
	Cost-inflicting mate-retention tactics	2.015	0.095	[1.828, 2.202]	1265.8	21.15	< .001
	Benefit-provisioning mate-retention tactics	-0.236	0.091	[-0.414, -0.058]	1280.8	-2.60	.009
	Age	-0.005	0.002	[-0.010, -0.000]	845.4	-2.05	.040
	Female vs. male	-0.157	0.058	[-0.270, -0.043]	856.1	-2.71	.007
	Minority ethnicity vs. White ethnicity	-0.069	0.065	[-0.196, 0.058]	800.1	-1.06	.289
	Relationship length: 6–12 months vs. < 6 months	-0.041	0.359	[-0.745, 0.662]	1271.0	-0.11	.909
	Relationship length: 1–2 years vs. < 6 months	-0.019	0.350	[-0.707, 0.668]	1265.7	-0.06	.956
	Relationship length: 2–3 years vs. < 6 months	-0.285	0.334	[-0.939, 0.370]	1274.1	-0.85	.393
	Relationship length: 3–5 years vs. < 6 months	-0.182	0.321	[-0.811, 0.448]	1297.0	-0.57	.571

Outcome	Predictor	B	SE	95% CI	df	*t*	*p*
	Relationship length: > 5 years vs. < 6 months	-0.214	0.308	[-0.817, 0.389]	1309.2	-0.70	.486
	Wave 1 × Brazil	0.267	0.412	[-0.543, 1.076]	541.7	0.65	.518
	Wave 1 × COVID-19 risk	0.084	0.085	[-0.083, 0.250]	592.1	0.99	.324
	Brazil × COVID-19 risk	-0.045	0.104	[-0.249, 0.158]	877.7	-0.44	.662
	Wave 1 × COVID-19 uncertainty	0.020	0.016	[-0.011, 0.051]	764.7	1.26	.207
	Brazil × COVID-19 uncertainty	0.002	0.018	[-0.034, 0.038]	928.1	0.11	.909
	Wave 1 × COVID-19 impact	-0.054	0.087	[-0.225, 0.118]	697.6	-0.62	.538
	Brazil × COVID-19 impact	-0.050	0.093	[-0.233, 0.133]	771.8	-0.54	.591
	Brazil × attachment anxiety	-0.090	0.070	[-0.228, 0.048]	1296.3	-1.28	.200
	Brazil × attachment avoidance	0.425	0.089	[0.250, 0.600]	1289.9	4.77	< .001
	Brazil × cost-inflicting tactics	-1.057	0.125	[-1.303, -0.810]	1285.5	-8.42	< .001
	Brazil × benefit-provisioning tactics	0.043	0.117	[-0.186, 0.272]	1276.4	0.37	.713
	Wave 1 × Brazil × COVID-19 risk	-0.070	0.112	[-0.291, 0.150]	610.9	-0.63	.531
	Wave 1 × Brazil × COVID-19 uncertainty	-0.023	0.020	[-0.062, 0.016]	677.4	-1.16	.246
	Wave 1 × Brazil × COVID-19 impact	0.092	0.102	[-0.109, 0.293]	660.0	0.90	.370

Table S4.2
Model Information

Statistic	Psychological IPV	Physical IPV	Sexual IPV
Observations	1400	1400	1400
Participants	1152	1152	1152
REML criterion	4405.3	3804.3	3794.9
Log-likelihood	-2202.6	-1902.2	-1897.5
AIC	4473.3	3872.3	3862.9
BIC	4651.6	4050.6	4041.2
Random-intercept variance	0.667	0.156	0.194
Residual variance	0.674	0.671	0.631
ICC	0.830	0.820	0.820

Note. B = unstandardised coefficient; SE = standard error; CI = confidence interval; ICC = intraclass correlation coefficient; AIC = Akaike information criterion; BIC = Bayesian information criterion. Models were adjusted for age, sex, ethnicity, and relationship length. Wave 2, the United Kingdom, male sex, White ethnicity, and relationship length below six months were the reference categories. Models were estimated using restricted maximum likelihood with Satterthwaite degrees of freedom and included a random intercept for participant. The covariance among repeated observations was modelled through the participant-level random intercept; residuals were assumed to be independent conditional on the random effect, with constant residual variance. Random-intercept and residual variance estimates were obtained from the final adjusted models. ICC values were obtained from unconditional intercept-only models.

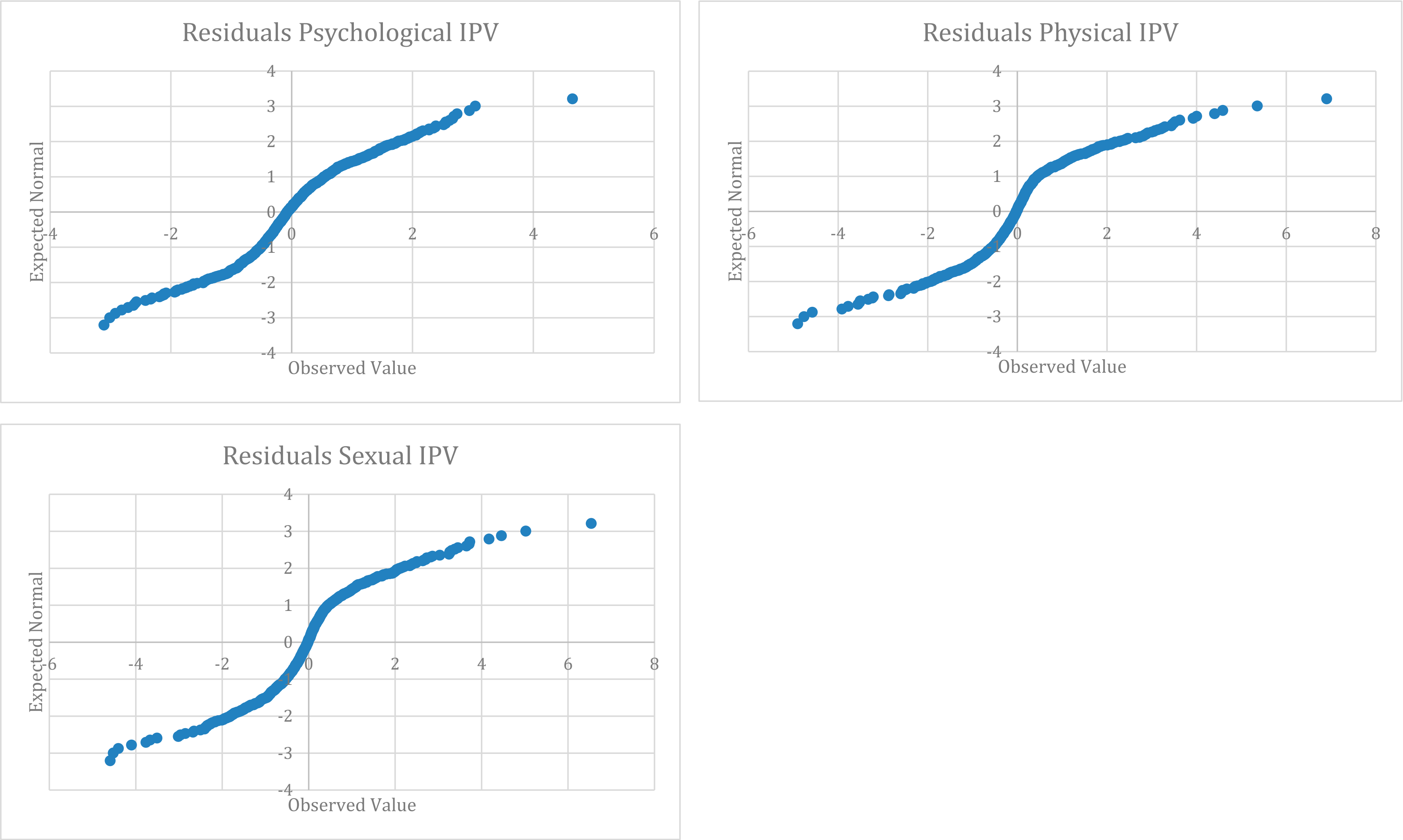

Figure S1. Normal Q-Q plots of residuals for Psychological, Physical, and Sexual IPV

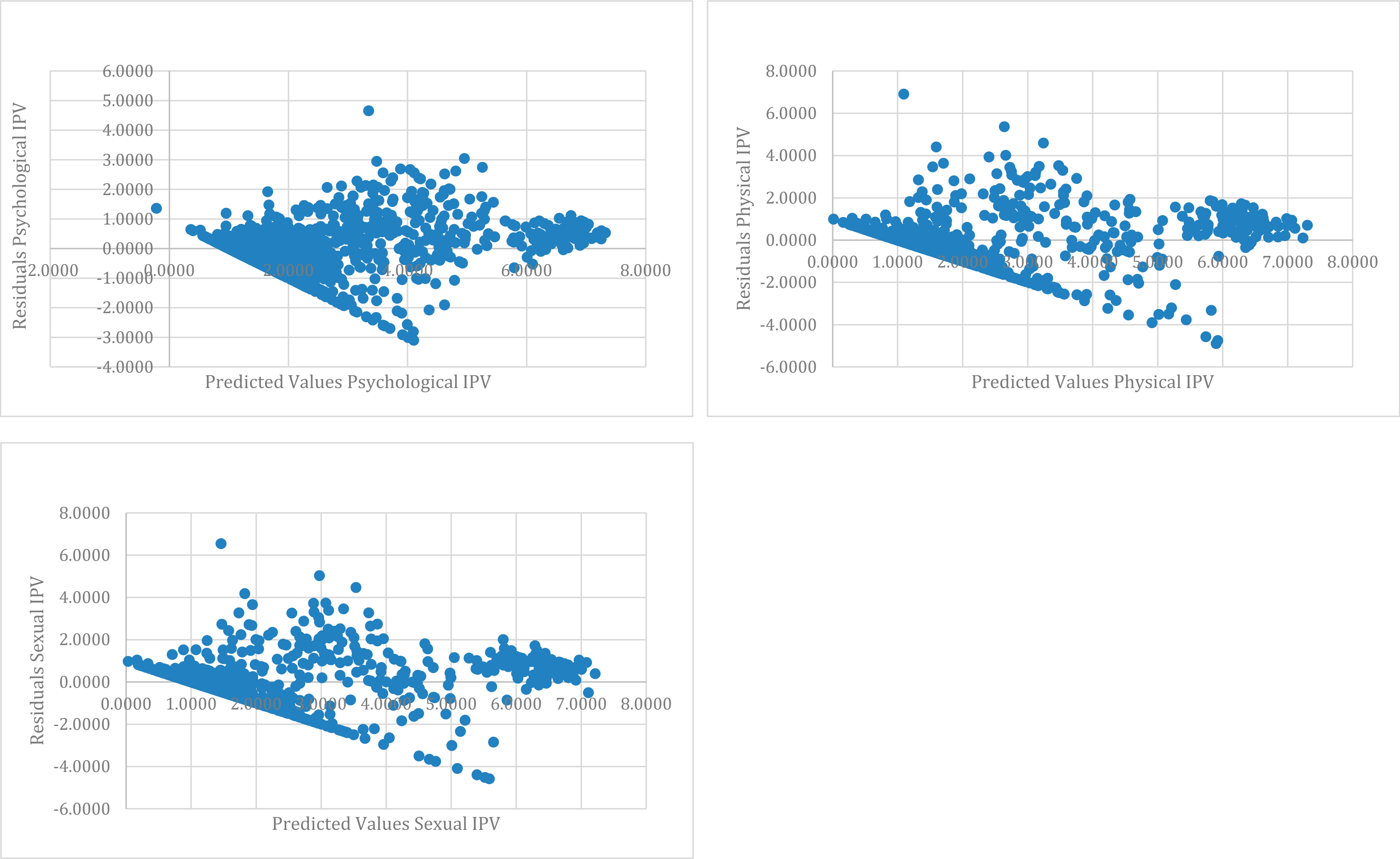

Figure S2. Residuals plotted against predicted values for the adjusted linear mixed-effects model predicting different forms of IPV

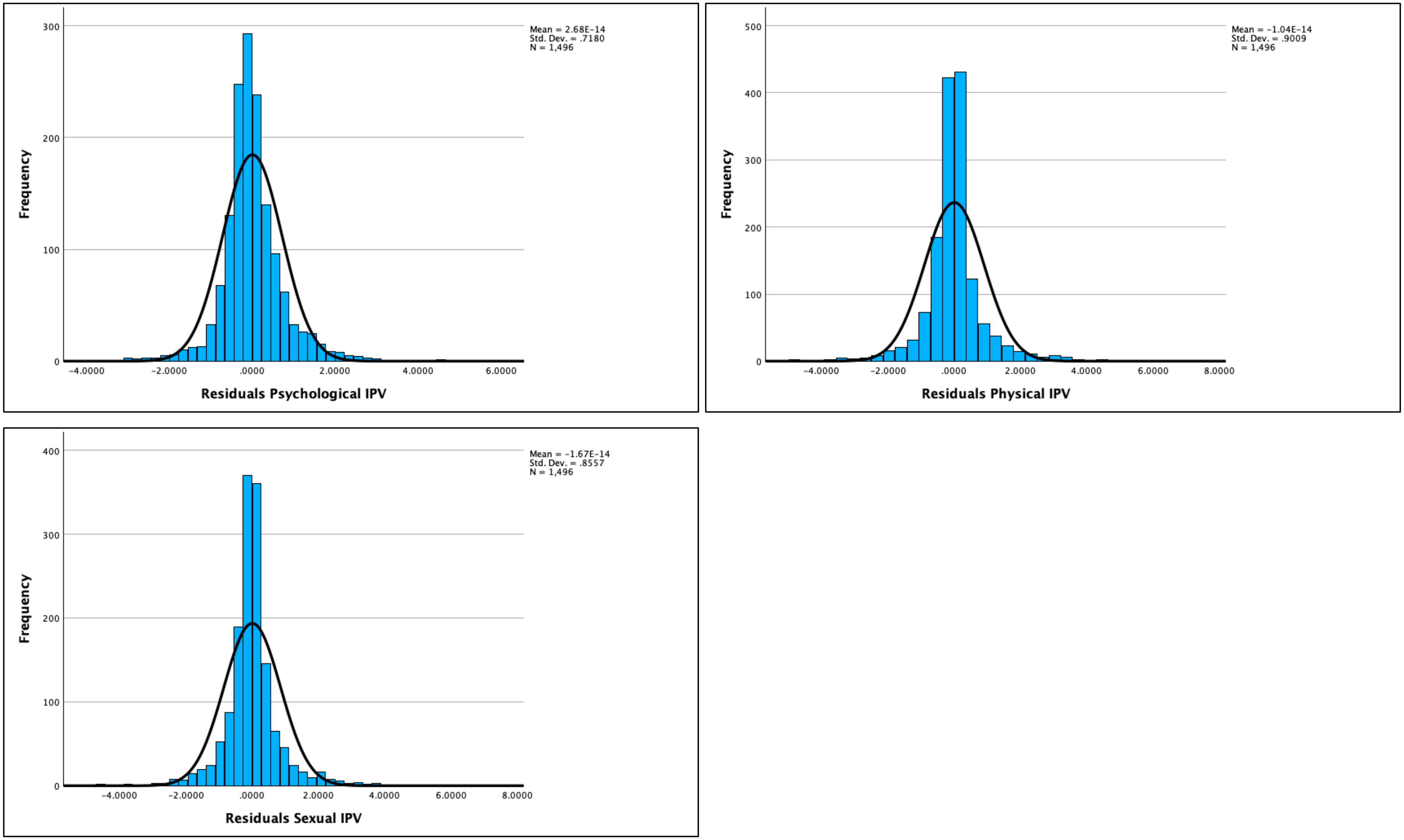

Figure S3. Distribution of residuals for the different IPV models
